# Life Satisfaction Among Individuals with Physical Disabilities in Saudi Arabia: The Impact of Physical Activity, Self-Perceived Health and Fitness, and Sociodemographic Features

**DOI:** 10.3390/medicina61010031

**Published:** 2024-12-28

**Authors:** Majed M. Alhumaid, Mohamed A. Said, Selina Khoo

**Affiliations:** 1 Department of Physical Education, College of Education, King Faisal University, Al-Ahsa 31982, Saudi Arabia; 2 Faculty of Sports and Exercise Science, Universiti Malaya, Kuala Lumpur 50603, Malaysia

**Keywords:** well-being, life satisfaction, sociodemographics, physical activity, physical disabilities

## Abstract

*Background and Objectives*: Life satisfaction (LS) is a key aspect of mental well-being, particularly for individuals with physical disabilities (IWPDs).This study examined LS levels among IWPDs in Saudi Arabia, focusing on the effects of three independent variables: (i) sociodemographic factors, (ii) self-reported health and fitness, and (iii) self-assessed physical activity (PA). *Materials and Methods:* Data was collected from 271 participants using validated questionnaires. Two models analyzed the effects of the independent variables on LS: the first model included the overall level of PA, while the second examined its individual components. *Results*: The results indicated that females reported a higher LS than males (*p* = 0.011). Participants with a university degree demonstrated a significantly greater LS compared to those who did not disclose their educational status (Exp(β) = 1.104). Poor health and inactivity were linked to a lower LS, while age correlated positively with LS (odds ratio = 1.012). Additional factors, including marital status, income, education level, and mobility assistance usage, significantly impacted LS. Interestingly, PA exhibited no direct statistical effect on LS. *Conclusions*: These findings highlight the importance of equitable access to education, regular PA, support for married couples, and preventive healthcare. Special attention to young people, particularly boys, is recommended to improve LS outcomes among IWPDs in Saudi Arabia.

## 1. Introduction

The importance of well-being in the establishment and sustainability of thriving and efficient communities has been widely acknowledged [1]. To keep an eye on people’s well-being, researchers use objective indicators of well-being, such as income, literacy, and life expectancy, as well as subjective assessments, such as how individuals perceive and experience life [1]. The method of assessing perceptions and life experiences is commonly referred to as subjective well-being (SWB). According to Diener [2], SWB can be defined as “a person feeling and thinking his or her life is desirable regardless of how others see it.” This definition highlights the thinking and feeling dimensions of SWB [3]. Thinking refers to the evaluative aspect of SWB, known as the evaluative/cognitive dimension. When individuals evaluate their lives predominantly in positive terms, it leads to a higher SWB. However, feeling encompasses both emotional and cognitive characteristics and is utilized to assess an individual’s level of life satisfaction (LS).

LS plays a vital role across all stages of life, but it is particularly significant in later life as it is closely linked to mental well-being [4]. Psychological needs, such as the desire for social affiliation and self-fulfillment, are essential to life satisfaction. When these needs are met, individuals tend to experience greater security, happiness, and usefulness, while unmet needs can lead to feelings of inadequacy [5]. Research has demonstrated that a higher LS is associated with improved mental health, including reduced levels of depression [3], while dissatisfaction with life can increase the risk of negative outcomes, including premature mortality [6].

The existing evidence indicates that individuals with disabilities have a lower level of LS compared to those without disabilities [7,8]. In their study, Nosek et al. [9] discovered that individuals with physical disabilities (IWPDs) had a mean LS score of 9.2 out of 20, which was significantly lower than the mean score of 13.2 obtained from a sample taken from the general population. In contrast, pertinent research indicates that IWPDs undergo favorable transformations linked to their impaired situations, such as an enhanced sense of gratitude for life, improved interpersonal connections, and increased resilience [10].

Research indicates also that a person’s personal LS can be significantly impacted by a range of conditions associated with disability or health. These factors include the severity of the disability [11], restrictions in functioning [12], chronic illnesses [7], and an individual’s subjective assessment of their health status [13]. Prior studies have also demonstrated that demographic factors such as gender, education, marital status, disability level, religion, and retirement planning are associated with a sense of LS among IWPDs [14]. Offering social assistance, whether through emotional or practical methods, to individuals with disabilities can mitigate their challenges and enhance their overall LS [7,13]. Furthermore, it has been shown that individuals with intellectual and developmental disabilities who actively participated in PA and sports displayed significantly elevated levels of LS compared to those who did not engage in such activities. An et al. [15] proposed that individuals of all age groups, ranging from young to older adults, who engaged in PA reported elevated levels of enjoyment and life satisfaction. In contrast, engaging in sedentary activity is associated with a negative correlation, which worsens the likelihood of acquiring mood disorders [16].

In Saudi Arabia, the prevalence of physical disabilities is estimated at 3.9% of the population, and these rates are expected to rise due to increasing health risk factors such as obesity and chronic diseases [17]. Additionally, data from the Saudi General Statistics Authority have shown a decline in the prevalence of PA among the general population, with rates dropping from 20.04% in 2019 to 29.7% in 2021. Regrettably, these statistics fail to consider individuals with disabilities. Furthermore, there is a dearth of research on PA among people with disabilities, and no study has explored the relationship between PA and LS in IWPDs, as far as we are aware.

To date, Zahra et al. [18] are among the few to offer studies that address the relationship between PA and well-being in both individuals with and without disabilities. Their study demonstrated a significant positive relationship between engaging in optimal PA and improved psychological well-being in individuals with disabilities. It also revealed that sedentary behavior negatively impacted the psychological quality of life in both disabled and non-disabled groups. Additionally, the study suggested that demographic factors, such as age and marital status, can influence the psychological well-being of individuals without disabilities, but no such association was found for people with disabilities.

Hence, this research endeavors to examine the self-perceived LS among IWPDs in Saudi Arabia. The study is guided by the following research inquiries:

1. What is the level of PA and LS among individuals with physical disabilities in Saudi Arabia?
2. Does a variation in LS level exist among participants based on demographic characteristics, self-assessed health, and self-assessed fitness?
3. Are demographic variables, PA, self-rated health, and self-rated fitness associated with LS?

## 2. Methods

### 2.1. Data Collection and Participants

This study used an online survey methodology to collect data. The sampling, participant recruitment, and data entry stages were carried out between 1 September and 31 December 2023. Recruitment was carried out among affiliates of the Riyadh-based Motor Handicapped Association for Adult Mobility and from three social rehabilitation centers located in the Eastern Province of Saudi Arabia. Eligible participants were individuals aged 18 years or older, literate, and with a confirmed diagnosis of a physical disability, verified through an official disability certification issued by the Saudi Ministry of Human Resources and Social Welfare. Additionally, participants were required to consent to participate in the study.

A total of 400 participants agreed to take part in the study, and a digital copy of the survey was emailed to each of them. The email included comprehensive information about the study’s objectives, methodology, inclusion criteria, and the incentive for participation. Participants were instructed to click on the hyperlink provided in the email, which directed them to the survey platform. On the initial page, they were required to review and sign a consent form. Before proceeding with the survey, participants were asked to carefully read and accept the stated conditions, ensuring informed and voluntary participation. Once they clicked “I Agree”, participants were taken to a three-part online survey on Google Drive. The estimated time required to complete the surveys was 15 min. After that, the results were downloaded (N = 277) and verified for accuracy. Six questionnaires were excluded for the following reasons: missing responses to key questions, including those related to life satisfaction (3 questionnaires); response patterns suggesting disinterest, such as consistently selecting the first option for all questions (1 questionnaire); and inconsistencies in demographic data, such as discrepancies between reported age and marital status (2 questionnaires). A total of 271 responses (males: n = 165; females: n = 106) were accepted for inclusion into the dataset (a completion rate of 67.65%).

### 2.2. Instruments

Data were collected using three questionnaires, comprising a total of 31 items. The first questionnaire, containing 13 items, gathered information on height, weight, self-rated health, self-rated physical fitness, and demographic characteristics. The second questionnaire, the Arabic version of the Physical Activity Scale for Individuals with Physical Disabilities (PASIPD-AR), included 13 items to evaluate participants’ PA levels and types of activities. The third questionnaire, Satisfaction with Life Scale (SWLS), consisted of 5 items and was used to measure life satisfaction.

#### 2.2.1. Demographic Questionnaire

This research instrument gathered data on participants’ age, gender, educational attainment, specific physical disabilities, type/s of physical assistive device/s employed, height, weight, marital status, occupation, and average family income, as well as their self-assessed health, and self-reported physical fitness. The latter two elements were assessed using a three-point Likert scale (bad, good, outstanding) based on the participant’s replies to two questions regarding their self-assessed health and self-reported physics fitness.

#### 2.2.2. PASIPD-AR

The PASIPD-AR is the Arabic adaptation of the Physical Activity Scale for Individuals with Physical Disabilities, originally developed by Washburn et al. [19]. This version was specifically tailored to the Saudi context, with its validity and reliability comprehensively evaluated by our research team [20]. The scale demonstrated robust construct validity through retrospective Confirmatory Factor Analyses (CFA) conducted on a sample of 206 individuals with physical disabilities. The factor loadings ranged from 0.583 to 0.889, explaining 61.62% of the total variance. A reliability analysis showed acceptable internal consistency, with a Cronbach’s alpha of 0.694 for the overall PASIPD-AR scale and subscale coefficients ranging from 0.42 to 0.619. These findings underscore the scale’s applicability and robustness for use within this context.

The PASIPD-AR comprises a total of 13 items that record the frequency of engagement in leisure activities, home activities, occupational activities, and inactivity over the course of the past week, specifying the number of days per week and hours per day. The PASIPD-AR differs from the original English version by including four latent factors instead of five. It includes the following: Factor 1: home repairs, lawn mowing, and garden work (items 9, 10, and 11); Factor 2: household activities (items 7, 8, and 12); Factor 3: sports and recreational activities (items 3, 4, 5, and 6); and Factor 4: occupational and transportation activities (items 2 and 13). The initial item is exempt from evaluation and functions as a reference. The participant is required to remember and report the frequency of engaging in various activities during the past 7 days. The frequency options are categorized as never/seldom (1–2 days/week), sometimes (3–4 days/week), or often (5–7 days/week). Additionally, the participant is requested to provide the average number of hours per day spent on these activities, with four options, including (1) less than 1 h, (2) more than 1 h but less than 2 h, (3) 2–4 h, and (4) more than 4 h. The daily hours for the occupational items are categorized as from less than 1 h to 8 h or more. The PASIPD-AR rating is determined by multiplying the mean daily duration of each activity by its corresponding metabolic equivalent (MET) value. The scores span from 0.0 MET h/day (indicating no activities completed) to 199.5 MET h/day (marking the longest duration of days and hours for all indicated activities performed).

#### 2.2.3. Satisfaction with Life Scale (SWLS)

The five-item SWLS was developed by Diener et al. [21] to assess LS globally via cognitive judgments rather than as a gauge of pleasant or negative effects. The participants express their degree of concurrence or dissent regarding each of the five assertions via a seven-point Likert scale that ranges from 7 (strongly agree) to 1 (strongly disagree). A concise summary of the five elements is as follows: Firstly, in most ways my life is close to my ideal. Secondly, the conditions of my life are excellent. Thirdly, I am satisfied with my life. Fourthly, so far, I have gotten the important things I want in life. Finally, if I could live my life over, I would change almost nothing. The score is maintained in a continuous fashion by aggregating the scores for each item. As benchmarks, the following cut-offs were employed: 31–35 for extremely satisfied, 26–30 for satisfied, 21–25 for slightly satisfied, 20 for neutral, 15–19 for slightly dissatisfied, 10–14 for dissatisfied, and 5–9 for extremely dissatisfied. Before collecting the data, the Arabic translation of the original English version was undertaken following the principles specified by Banville et al. [22]. A pilot study was conducted on 17 IWPDs. The Cronbach’s alpha coefficient was found to be 0.842, while the R-values were 0.792, 0.845, 0.829, 0.815, and 0.646, respectively.

### 2.3. Data Analysis

To address the first two research objectives, we calculated the frequency of each LS category, the degree of PA and categories, and the mean and standard deviation of the LS in all participants based on the independent variables. The statistical methods used to evaluate the significance differences across subgroups were t-tests and a one-way ANOVA with post hoc tests (specifically, Tukey HSD). The final research objective was analyzed using Pearson correlation analysis to determine the strength of the association between PA, self-rated health, self-rated fitness, demographic variables, and LS among IWPDs. The following thresholds were used to interpret the strength of the relationships between variables: strong correlation (r > 0.5 or r < −0.5), moderate correlation (r between 0.3 and 0.5, or between −0.3 and −0.5), and weak correlation (r < 0.3 or r > −0.3; [23]. Additionally, ordinal logistic regression (OLR) was employed to assess the impact of the independent variables on LS. LS classifications were utilized as an ordinal variable, with BMI and PA serving as covariates, while the remaining independent variables were considered factors. In order to mitigate the confounding impact, two models were executed, one incorporating the PASIPD-AR score and the other incorporating its individual components. The results were presented as unstandardized beta coefficients (β) with standard errors, Wald χ^2^ statistics, Exp(β) values, and 95% confidence intervals (CIs). Beta (β) coefficients indicate the change in the Log odds of LS for a one-unit change in the predictor, while Exp(β) (odds ratio) reflects the relative change in the odds of LS. Exp(β) > 1 indicates increased likelihood, while Exp(β) < 1 indicates decreased likelihood. Confidence intervals for Exp(β) provide precision for these estimates. The assumptions for the analyses were evaluated with specific tests using SPSS 26 (IBM, Armonk, NY, USA), and the significance threshold was set at *p* < 0.05.

### 2.4. Ethical Considerations

The study was conducted in accordance with the Declaration of Helsinki and approved by the Research Ethics Committee of King Faisal University, Saudi Arabia, (KFU-REC-2023-JUN-ETHICS1091). The ethical approval committee carried out a full evaluation of the online survey hosted on Google Drive, and the nature of the consent was deemed acceptable.

## 3. Results

The sample (N = 271) primarily consisted of males (69.9%). Among the participants, 25.1% had a spinal disease, 8.9% had progressive muscular dystrophy, 10.0% had sclerosis multiplex, 13.3% had cerebral palsy, 14.0% had poliomyelitis, 7.7% had a leg or foot amputation, and 21.0% had other disabilities. Participants’ characteristics are summarized in Table 1. The average age of the participants was 39.36 ± 11.74 years old. Their average BMI was 27.95 ± 7.6 kg·m^−2^, and their PASIPD-AR score was 15.22 ± 27.69 MET h/day. The highest score for the specific components of the PASIPD-AR was observed in occupational and transportation activities (4.74 ± 4.92 MET h/day). However, the lowest score was noted in home repairs, lawn mowing, and garden works (2.06 ± 7.53 MET h/day). The mean satisfaction with life score was 19.00 ± 8.05, indicating a general state of mild discontent among the participants. Specifically, 17% expressed extreme dissatisfaction, 11.8% were dissatisfied, 22.1% were slightly dissatisfied, 4.4% had a neutral opinion, 21% were slightly satisfied, 15.1% were satisfied, and 8.5% were highly satisfied.

Table 2 displays the mean level of LS among participants based on their demographic characteristics, self-rated health, self-rated fitness, and use of mobility assistive devices. Females reported significantly greater levels of LS compared to males (t = −2.568; *p* = 0.011). Single participants reported lower levels of LS compared to divorced individuals (ΔM = −7.573; *p* = 0.004) and those who opted not to disclose their marital status (ΔM = −6.76; *p* = 0.04). Individuals with a university degree reported higher levels of LS compared to those with only a primary or high school degree (ΔM = 7.062 and 6.217; *p* < 0.001 for both groups). Employed individuals reported higher levels of LS compared to the unemployed (ΔM_public_ = 4.855; ΔM_private_ = 3.977; *p* = 0.004 for both sectors). No noticeable differences were evident among participants’ self-reported LS in relation to average household income. Except for those with progressive muscular dystrophy, participants with cerebral palsy reported significantly lower levels of LS compared to those with spinal disease, SM, poliomyelitis, leg or foot amputation, and Others (ΔM = −6.389; *p* = 0.013 for SM; ΔM = −8.314, −12.039, −10.881, and −6.781, respectively; *p* < 0.001 for all). There were also notable differences in participants’ self-reported LS between those with progressive muscular dystrophy and poliomyelitis (ΔM = −6.577; *p* = 0.012), as well as between those with poliomyelitis and Others (ΔM = 5.254; *p* = 0.013). In terms of self-assessed health and self-reported PA, significant statistical differences were evident among particular subgroups. For example, those who assessed their health more favorably reported having significantly higher levels of LS compared to those who rated their health more poorly (ΔM = −7.495 and −10.955; *p* < 0.001 for good and excellent health, respectively; ΔM = −3.461; *p* = 0.01 for good vs. excellent). In addition, those who identified themselves as moderately active or active/extremely active reported higher levels of LS compared to those who were entirely inactive (ΔM = 5.94 and 9.737; *p* < 0.001 for both). Ultimately, those who use canes for their mobility enjoy higher levels of LS compared to those who do not use any mobility aids (ΔM = 6.722; *p* = 0.001), or those who depend on wheelchairs (ΔM = 4.854; *p* = 0.006) or crutches (ΔM = 4.691; *p* = 0.05).

### 3.1. Correlation Analysis

Pearson correlation was used to investigate the effect of demographic characteristics, self-rated health, self-reported fitness, mobility assistance device use, and measured PA on overall LS as well as its five items. Except for BMI, type of disability, and occupational and transportation activities, all independent variables positively correlated with the overall LS of Saudi people with physical disabilities. The correlations were moderate for self-rated health and self-rated physical fitness and weak for the remaining variables (Table 3). No significant relationship was noted between the LS items and BMI, type of disability, and occupational and transportation activities. Regarding the LS components, the items “I am satisfied with my life” and “The conditions of my life are excellent” showed moderate positive correlations with self-rated health (r = 0.385 and 0.422, respectively). Additionally, “I am satisfied with my life” exhibited a moderate correlation with self-rated physical fitness (r = 0.315). A moderate relationship was also observed between the item “So far, I have gotten the important things I want in life” and the educational level of participants (r = 0.314). The remaining significant associations were positive but weak (Table 3).

### 3.2. Regression Analysis

An OLR analysis was performed to identify the demographic parameters, self-rated health, self-reported fitness, mobility assistance device use, and measured PA that had a significant impact on the LS of IWPDs in Saudi Arabia. Two models were conducted. In the first model, the PASIPD-AR score was used, while in the second model, the PA components were incorporated to reduce any possible confounding effect.

The assumption of the OLR was satisfied, as evidenced by a Chi-square value of 142.615 and a *p*-value of 0.078. Furthermore, the model exhibited a satisfactory fit to the data, and the McFadden value indicated a significant (19.8%) enhancement in LS, as compared to the null model, based on the predictors. Both models concluded that there was no statistically significant effect of PA on LS (Table 4). However, the findings indicated that age is positively correlated with life satisfaction (LS) (Wald χ^2^ = 6.325, *p* = 0.012), with a β value of 0.035, suggesting that as age increases, participants are more likely to report a higher LS. For each additional year of age, LS increases by approximately 3.6% (Exp(β) = 1.036, 95% CI [1.008, 1.064]).

Additionally, several factors were found to have a substantial impact on LS:

- Educational attainment (β = 1.337): Participants with a university degree reported a 280.8% higher LS compared to those with a postgraduate degree (Exp(β) = 3.808, 95% CI [1.069, 13.572], Wald χ^2^ (1) = 4.256, *p* = 0.039);
- Self-rated health (β = −2.335): Participants who rated their health as poor experienced a 90.3% decrease in LS compared to those who rated their health as excellent (Exp(β) = 0.097);
- Self-reported fitness (β = −1.365): Participants who reported poor fitness had a 74.5% lower LS compared to those who reported good or excellent fitness (Exp(β) = 0.255);
- Use of mobility assistance devices (β = 1.315): Individuals using canes for mobility reported a 272.5% higher LS compared to those relying on crutches (Exp(β) = 3.725, 95% CI [1.436, 9.670], Wald χ^2^ (1) = 7.309, *p* = 0.007).

The OLR analysis also revealed significant gender differences in LS. Males exhibited a 52.2% decrease in LS compared to females (Exp(β) = 0.478, 95% CI [0.276, 0.824], Wald χ^2^ (1) = 7.037, *p* = 0.008).

Marital status was another significant factor. Participants who were single or widowed reported an 81.6% and 84.1% lower LS, respectively, compared to those who did not disclose their marital status.

Finally, family income had a notable effect. Individuals from families with a monthly income of less than 5000 Saudi riyals experienced a 63.7% decrease in LS compared to those who chose not to disclose their family income (Exp(β) = 0.363, 95% CI [0.146, 0.902], Wald χ^2^ (1) = 4.759, *p* = 0.029).

## 4. Discussion

The purpose of this research was to look at how physically disabled people in Saudi Arabia rate their own satisfaction with life. The Physical Activity Scale for Individuals with Physical Disabilities—Arabic version—was used to evaluate the amounts and kinds of PA performed by the participants. The next step was to study the connections between IWPDs in Saudi Arabia’s demographics, self-assessed health, fitness, mobility aid use, and PA with LS.

The average LS score was 19 ± 8.05 out of a maximum potential score of 35, equivalent to approximately 54%. The survey results indicate that 17% of the participants were extremely dissatisfied, 11.8% were dissatisfied, 22.1% were slightly dissatisfied, 4.4% had a neutral attitude, 21% were slightly satisfied, 15.1% were satisfied, and 8.5% were highly satisfied. The results indicated that the participants had a low level of LS overall. Regrettably, no prior studies have comprehensively examined the LS of IWPDs in Saudi Arabia, and so we are unable to compare our results with previous research. The present study represents an initial attempt to investigate this topic. That said, a study by Alsubaie [24] on a sample of 453 adolescents without disabilities aged 15–20 years old found that 21.7% were relatively satisfied with their lives (either extremely satisfied or satisfied). Conversely, 78.3% reported being dissatisfied with their lives. Relatedly, a study by El Keshky et al. [25] found that LS among 260 healthy participants (142 women and 118 men with an average age of 65.3 years) was 23.13 ± 5.7 using the same measurement scale as the present study.

Prior research indicates that individuals without disabilities report higher levels of LS compared to those with disabilities. Pans et al. [26] found that IWPDs typically reported lower levels of LS compared to those without disabilities among a sample of 1227 university students. The results indicated a median LS score of 16, with an interquartile range of 20. These values align with a degree of LS classified as somewhat dissatisfied. A recent longitudinal study by Nicolaisen et al. [27] analyzed Norwegian panel data on 2555 individuals aged 40–79 to investigate the impact of disability and its duration on LS after a follow-up period of five years. Individuals without disabilities had significantly higher SWLS scores (M = 19.5, SD = 3.1) compared to those with a long-term disability (M = 18.0, SD = 3.7; *p* < 0.001), those with a short-term disability (M = 18.8, SD = 3.1; *p* < 0.002), and those who had previously had a disability but no longer did (M = 18.9, SD = 3.5; *p* < 0.019).

The impact of individual factors on LS in those without disabilities is covered extensively in the literature. Much less research exists, however, on the impact of individual factors on the LS of IWPDs. One key finding of the current study is the gender-based differences in LS among IWPDs: females were more likely to report higher levels of LS than males (20.55 ± 7.65 vs. 18.00 ± 8.17). This concurs with Emerson and Llewellyn’s [28] results: the overall prevalence of a high LS was 55.5% in women and 48.0% in men. Interestingly, Montgomery [29] investigated the reasons for women’s higher happiness levels despite their often poor educational level, income, health status, and possibilities. By incorporating anchor vignettes based on data from the Gallup World Poll across 102 countries, the author demonstrated that males and females consistently employ distinct response scales, resulting in women reporting to be less happy than men on average when these scales are standardized. After adjusting the vignettes, the impacts of other often researched attributes such as wealth, education, and marital status remained consistent in direction, thereby supporting the prior findings.

Meanwhile, Buecker et al. [30] found a statistically significant positive correlation between LS and age: LS decreases between the ages of 9 and 16, rises marginally until the age of 70, and then declines once more until the age of 96. This is of particular interest to us given the age range of the participants in the present study (18–64); it may be that as IWPDs age, the more determined they become to adapt to and accept the limitations imposed by their disability and thus develop ways to improve their LS. In the elderly, health and mobility issues are regarded as “normal” and unavoidable. LS can be safeguarded and preserved as individuals age through acceptance and adjustment to declining health and other concomitantly negative life circumstances.

Much scholarly work indicates that IWPDs report higher levels of marital dissatisfaction and depressive symptoms compared to their non-disabled peers [14]. Marital dissatisfaction was a contributing factor to the psychological distress, burdensome responsibilities, and lack of family support experienced by spouses of individuals with disabilities [31]. The results of our study indicate that divorced individuals have the highest level of LS, although LS declined in widowers compared to those who preferred not to declare their marital status.

The findings revealed two key results: First, all components of life satisfaction showed significant correlations with self-rated physical fitness, suggesting that individuals who perceive themselves as physically fit generally report higher life satisfaction across various domains [15]. Second, a significant association was found between self-rated health and nearly all dimensions of life satisfaction, highlighting the crucial role of health perceptions in overall well-being [32]. These results underscore the interconnectedness of physical health, fitness, and life satisfaction, pointing to the need for further, more in-depth investigation into these relationships.

Strong evidence supports the importance of PA in improving LS among IWPDs [14,26,33]. LS tends to be higher in those who engage in PA and are committed to exercise and sports [15]. The results suggest that individuals who participate in PA report higher levels of LS compared to those who do not. A strong correlation between LS and PA exists in both adults and adolescents [15,34]. The present study supports the positive relationship between PA and LS in IWPDs. However, the PASIPD-AR score did not show a direct positive effect on LS, with only self-rated physical fitness having a significant and favorable impact. Individuals who reported being inactive had a 74.5% lower LS compared to those who were active or extremely active (Exp(β) = 0.255).

Consistent with our research, Martin Ginis et al. [35] confirmed that engaging in sports and PA is linked to minor alterations in the SWB of individuals with disabilities. Nevertheless, they emphasized the significance of differentiating between performance-related factors (such as frequency, intensity, and time spent) and experiencing factors of engaging in sports and exercise for those with disabilities. According to Kim et al. [14], specific forms of recreational activities have been found to improve health, enhance LS, and promote positive attitudes towards disability. The significance of social engagement in enhancing the quality of life for older individuals with physical disabilities was emphasized. Engaging in social activities can improve mental well-being, increase LS, and positively influence one’s impression of health. Engaging in activities has been utilized to establish social bonds and foster a feeling of belonging to a group, which is associated with psychological well-being [36].

Our findings also revealed a substantial correlation between LS and family income. Several studies suggest that financial status is the main situational factor that affects the LS of individuals with disabilities [27]. Having a disability can significantly impact one’s quality of life and financial status, especially when such individuals retain high expectations for their lives. An individual’s financial circumstances play a crucial role in obtaining material possessions for oneself and one’s family and serve as indicators of success and social standing. Inadequate material resources for handling life problems and responsibilities can be especially challenging during this life stage, and individuals with disabilities in this age bracket may not have fully adjusted to their circumstances. The WHO asserted that assistive technologies have the potential to enhance the lives of individuals with disabilities across various domains, including education, employment, physical fitness, leisure, and daily tasks such as personal care, cooking, and reading. This assistive technology can yield favorable outcomes for an individual, their family, and acquaintances, and it has broader socio-economic advantages. Providing suitable wheelchairs, for instance, enables people to move about more easily. This improves their ability to attend school or work, while also decreasing healthcare expenses by preventing secondary issues like pressure sores and contractures. Providing assistive technologies to individuals with disabilities in a timely manner can enhance their autonomy and security, allowing them to reside in their homes for an extended period [37].

Finally, our findings corroborated those of other research indicating that a higher level of education appears to be associated with a greater LS among IWPDs [38]. Di Tella et al. [39] found similar results by analyzing psychological data from Europe and the USA, showing that higher levels of education have a positive impact on self-reported LS. Frey and Stutzer [40] discovered a positive correlation between educational attainment and LS that remained significant even after accounting for variables like income and health, which are mechanisms via which education might influence LS. Blanchflower [41] showed that individuals with a greater educational attainment are generally more content with their lives.

Despite its merits, this study has several limitations. First, the cross-sectional approach used collects data at a single point in time, which limits the ability to establish causal relationships between variables and reduces the generalizability of the findings to other time periods or populations. Longitudinal studies are needed to assess the directionality and causality of the observed correlations [42]. Second, the influence of factors such as aging, depression, and social support perception on LS in IWPDs was not examined [27]. Future research is required to examine the disparities in depression and the perceptions of social support in IWPDs with LS across different age groups. Third, this study did not include information on when participants first developed their disability: the duration over which a physical disability develops can impact the quality of life of the individual involved. Therefore, future studies should investigate the correlation between the duration of a disability and LS of the individual involved. Fourth, the assessment of PA using self-reported questionnaires could be a source of bias and inaccuracies. For instance, some respondents may deliberately exaggerate their level of PA to align with social desirability norms [43]. Using direct measures of PA, such as pedometers or accelerometers, would enhance objectivity and accuracy [44]. Similarly, self-reported measures of body composition, health status, and physical fitness have inherent limitations in both the objectivity and accuracy of the findings. Employing direct tools—such as stadiometers, scales, skinfold calipers, dual-energy X-ray absorptiometry (DEXA), or bioimpedance analyzers for body composition; medical reports for health status; and specific physical tests for physical fitness—could provide more reliable and valid data, thereby strengthening the findings. Lastly, although all subjects in the present study were classified as IWPDs, they exhibited varying functional capacities. IWPDs with strong functional abilities may experience increased happiness [13]; therefore, investigating the correlation between functional ability and LS may offer healthcare providers valuable insights.

## 5. Implications and Conclusions

The results have five implications for enhancing LS among Saudi IWPDs. First, the participants’ level of education significantly influenced their LS; however, statistics from the Saudi Ministry of Education indicate that many IWPDs either do not attend school or are compelled to drop out. Therefore, it is recommended that all IWPDs be granted equal access to appropriate educational opportunities in order to improve their level of LS. In addition, providing alternative options for continuous learning is strongly advised for IWPD who are currently not enrolled in formal education. Secondly, income has a substantial impact on the LS of Saudi IWPDs. Therefore, implementing structured job placements and training initiatives for IWPDs is crucial to help enhance their LS. Third, marital status significantly predicts LS in IWPDs. However, many Saudi IWPDs encounter marital difficulties because of the physical and economic constraints imposed on them due to their condition, leading to a reduced quality of life [31]. Therefore, there is a need for the provision of family therapists and counselors to offer specialized programs to help IWPDs address their issues and receive solutions and assistance to enhance their marital relationships [14]. Fourth, it is imperative that IWPDs increase their involvement in PA as a result of the advantageous impact that PA has on their overall LS. Participating in activities such as home repairs, lawn mowing, and gardening, as well as household chores and sports and recreational activities, is a useful way to improve one’s LS. It also provides an opportunity to have healthy social connections with other family members. Fifth, as self-perceived health ratings are linked to positive physical health characteristics, good mental health, and optimal cognitive functioning [45], and can therefore improve LS among IWPDs, both governmental and non-governmental Saudi organizations should seek to enhance the physical and mental well-being of the community by offering regular healthcare provision, targeted interventions, and treatment strategies for IWPDs.

## Figures and Tables

**Table 1:** Participant characteristics.

	Min.	Max.	Mean	Std. Deviation
Age	18.00	64.00	39.36	11.74
Weight (kg)	37.00	130.00	71.58	18.35
Height (cm)	99.00	189.00	160.75	12.35
BMI (kg/m^2^)	13.84	49.95	27.95	7.60
Home repairs, lawn mowing, and garden works (MET h/day)	0.00	51.48	2.06	7.53
Household activities (MET h/day)	0.00	33.52	3.28	6.04
Sports and recreational activities (MET h/day)	0.00	87.95	4.32	12.22
Occupation and transportation activities (MET h/day)	0.00	75.00	4.74	7.92
PASIPD-AR score (MET h/day)	0.00	199.46	15.22	27.69
Life satisfaction	5.00	35.00	19.00	8.05

Note: The life satisfaction scores range from 5 to 35.

**Table 2:** Life satisfaction according to sociodemographics.

			SWLS Score			
		N	Mean	SD	t/f	*p*
Gender	Male	165	18.00	8.17	−2.568	0.011
	Female	106	20.55	7.65		
Marital status	Single	100	16.99	7.47	4.878	0.001
	Married	128	19.45	8.46		
	Divorced	16	24.56	9.27		
	Widowed	15	18.80	3.30		
	Don’t want to respond	12	23.75	4.63		
Educational level	Primary school degree	49	16.33	8.33	9.210	<0.001
	Middle school degree	31	19.00	8.44		
	High school degree	111	17.17	7.27		
	University degree	67	23.39	7.58		
	Postgraduate degree	13	22.00	4.93		
Occupation	Not working	186	17.65	8.23	8.972	<0.001
	Government employee	32	22.50	6.72		
	Private sector employee	53	21.62	6.91		
Average family income	<5000 SR	159	18.16	8.28	2.483	0.061
	5000 to 10,000 SR	80	19.52	7.65		
	>10,000 SR	13	23.77	6.61		
	Don’t want to response	19	20.53	7.67		
Type of disability	Spinal disease	68	20.15	8.42	9.938	<0.001
	Progressive muscular dystrophy	24	17.29	7.06		
	SM	27	18.22	9.61		
	Cerebral palsy	36	11.83	5.12		
	Poliomyelitis	38	23.87	5.61		
	Leg or foot amputation	21	22.71	7.22		
	Others	57	18.61	7.14		
Self-rated health	Poor	36	11.83	7.50	23.546	<0.001
	Good	183	19.33	7.48		
	Excellent	52	22.79	7.30		
Self-rated physical fitness	Not active at all	84	14.63	8.03	23.028	<0.001
	Moderately active	168	20.57	7.17		
	Active/extremely active	19	24.37	7.40		
Mobility assistive device	No	54	16.83	7.29	5.476	0.001
	Yes, wheelchair	144	18.70	8.42		
	Yes, cane	36	23.56	7.16		
	Yes, crutches	37	18.86	6.95		

**Table 3:** Life satisfaction correlations in individuals with physical disabilities.

	In Most Ways My Life Is Close to My Ideal	The Conditions of My Life Are Excellent	I Am Satisfied With My Life	So Far, I Have Gotten the Important Things I Want in Life	If I Could Live My Life Over, I Would Change Almost Nothing	SWLS
Gender	0.153 *	0.164 **	0.110	0.070	0.112	0.188 **
BMI (kg/m^2^)	0.104	0.076	0.077	0.097	0.101	0.094
Marital status	0.244 ***	0.176 **	0.203 **	0.079	0.106	0.215 ***
Age (categories)	0.256 ***	0.194 **	0.131 *	0.158 **	0.071	0.239 ***
Educational level	0.102	0.267 ***	0.250 ***	0.314 ***	0.085	0.212 **
Occupation	0.163 **	0.245 ***	0.288 ***	0.119	0.074	0.206 **
Average family income	0.070	0.197 **	0.174 **	0.055	−0.015	0.144 *
Type of disability	0.005	0.044	0.053	0.048	−0.031	0.035
Self-rated health	0.264 **	0.422 ***	0.385 ***	0.279 ***	0.115	0.350 ***
Self-rated physical fitness	0.390 ***	0.286 ***	0.315 ***	0.271 ***	0.236 ***	0.379 ***
Mobility assistive device	0.030	0.131 *	0.090	0.186 **	0.131 *	0.131 *
Home repairs, lawn mowing, and garden works	0.225 ***	0.153 *	0.118	0.149 *	−0.055	0.147 *
Household activities	0.228 ***	0.089	0.112	0.167 **	0.069	0.140 *
Sports and recreational activities	0.237 ***	0.053	0.055	0.211 ***	−0.033	0.164 **
Occupational and transportation activities	0.102	0.068	0.111	0.104	0.037	0.078
PASIPD-AR	0.258 ***	0.103	0.097	0.241 ***	0.068	0.193 **

Note: * *p* < 0.050, ** *p* < 0.01, *** *p* < 0.001.

**Table 4:** Ordinal logistic regression predicting overall life satisfaction in individuals with physical disabilities.

							95% CI	
		β	SE	Wald	Sig.	Exp(β)	LB	UB
Threshold	[SWL (Extremely dissatisfied)]	−4.701	1.255	14.023	0.000			
	[SWL (Dissatisfied)]	−3.708	1.245	8.875	0.003			
	[SWL (Slightly dissatisfied)]	−2.355	1.237	3.625	0.057			
	[SWL (Neutral)]	−2.104	1.236	2.897	0.089			
	[SWL (Slightly satisfied)]	−0.705	1.231	0.328	0.567			
	[SWL (Satisfied)]	0.907	1.233	0.542	0.462			
Location	Age	0.035	0.014	6.325	0.012	1.036	1.008	1.064
	BMI	−0.002	0.017	0.012	0.913	0.998	0.966	1.031
	PASIPD-AR	0.086	0.116	0.548	0.459	1.090	0.868	1.368
	[Gender (Male)]	−0.739	0.279	7.037	0.008	0.478	0.276	0.824
	[Gender (Female)]							
	[Marital Status (Single)]	−1.694	0.668	6.433	0.011	0.184	0.050	0.680
	[Marital Status (Married)]	−1.204	0.619	3.788	0.052	0.300	0.089	1.009
	[Marital Status (Divorced)]	0.342	0.775	0.195	0.659	1.408	0.309	6.424
	[Marital Status (Widowed)]	−1.836	0.775	5.615	0.018	0.159	0.035	0.728
	[Marital Status (Chose Not to Respond)]							
	[Educational (Primary Degree)]	0.108	0.682	0.025	0.875	1.114	0.293	4.238
	[Educational (Middle Degree)]	0.377	0.704	0.288	0.592	1.458	0.367	5.795
	[Educational (High Degree)]	0.094	0.620	0.023	0.880	1.099	0.326	3.699
	[Educational (University Degree)]	1.337	0.648	4.256	0.039	3.808	1.069	13.572
	[Educational (Postgraduate)]							
	[Occupation (Not Working)]	−0.530	0.321	2.730	0.098	0.589	0.314	1.104
	[Occupation (Government Employee)]	−0.539	0.481	1.257	0.262	0.583	0.227	1.498
	[Occupation (Private Employee)]							
	[Income (<5000 SR)]	−1.013	0.464	4.759	0.029	0.363	0.146	0.902
	[Income (5000–10,000 SR)]	−0.769	0.503	2.333	0.127	0.463	0.173	1.244
	[Income (>10,000 SR)]	−0.200	0.695	0.083	0.773	0.819	0.210	3.196
	[Income (Chose Not to Respond)]							
	[Self-Rated Health (Poor)]	−2.335	0.522	20.040	0.000	0.097	0.035	0.269
	[Self-Rated Health (Good)]	−0.640	0.358	3.200	0.074	0.527	0.262	1.063
	[Self-Rated Health (Excellent)]							
	[Self-Rated fitness (Poor)]	−1.365	0.563	5.872	0.015	0.255	0.085	0.770
	[Self-Rated fitness (Good)	−0.684	0.511	1.791	0.181	0.505	0.185	1.374
	[Self-Rated fitness (Excellent)							
	[Mobility Assistive Device (No)]	−0.080	0.426	0.036	0.850	0.923	0.400	2.128
	[Mobility Assistive Device (Wheelchair)]	0.275	0.378	0.529	0.467	1.317	0.628	2.759
	[Mobility Assistive Device (Cane)]	1.315	0.487	7.309	0.007	3.725	1.436	9.670
	[Mobility Assistive Device (Crutches)]							

## Data Availability

The data that support the findings of this study are available from the authors, upon reasonable request.

## References

[1] Durand M. (2015). The OECD better life initiative: How’s life? and the measurement of well-being. Rev. Income Wealth.

[2] Diener E. (2009). The Science of Well-Being: The collected Works of Ed Diener.

[3] Das K.V., Jones-Harrell C., Fan Y., Ramaswami A., Orlove B., Botchwey N. (2020). Understanding subjective well-being: Perspectives from psychology and public health. Public Health Rev..

[4] Sánchez-Rodríguez Á., Vignoles V.L., Bond M.H., Adamovic M., Akotia C.S., Albert I., Appoh L., Baltin A., Barrientos P.E., Denoux P. (2023). Self-construals predict personal life satisfaction with different strengths across societal contexts differing in national wealth and religious heritage. Self Identity.

[5] Ryan R.M., Deci E.L. (2017). Self-Determination Theory: Basic Psychological Needs in Motivation, Development, and Wellness.

[6] Pedišić Ž., Greblo Z., Phongsavan P., Milton K., Bauman A.E. (2015). Are total, intensity- and domain-specific physical activity levels associated with life satisfaction among university students?. PLoS ONE.

[7] Lin C.Y., Cheng T.C. (2019). Health status and life satisfaction among people with disabilities: Evidence from Taiwan. Disabil. Health J..

[8] Li P.S., Hsieh C.J., Shih Y.L., Lin Y.-T., Liu C.-Y. (2023). The effect of research on life satisfaction in middle-aged and older adults: Physical disability and physical activity as a parallel and serial mediation analysis. BMC Geriatr..

[9] Nosek M.A., Young M.E., Rintala D.H., Howland C.A., Foley C.C., Bennett J.L. (1995). Barriers to reproductive health maintenance among women with physical disabilities. J. Women’s Health.

[10] Kim J., Kim M., MaloneBeach E.E., Han A. (2016). A study of health perception, disability acceptance, and life satisfaction based on types of leisure activities among Koreans with a physical disability. Appl. Res. Qual. Life.

[11] Smith D.L. (2015). Does type of disability and participation in rehabilitation affect satisfaction of stroke survivors? Results from the 2013 Behavioral Risk Surveillance System (BRFSS). Disabil. Health J..

[12] Devereux P.G., Bullock C.C., Gibb Z.G., Himler H. (2015). Social-ecological influences on interpersonal support in people with physical disability. Disabil. Health J..

[13] Addabbo T., Sarti E., Sciulli D. (2016). Disability and Life Satisfaction in Italy. Appl. Res. Qual. Life.

[14] Kim J., Lee C., Ji M. (2018). Investigating the domains of life satisfaction in middle-aged, late middle-aged, and older adults with a physical disability. J. Dev. Phys. Disabil..

[15] An H.Y., Chen W., Wang C., Yang H.-F., Huang W.-T., Fan S.-Y. (2020). The Relationships between Physical Activity and Life Satisfaction and Happiness among Young, Middle-Aged, and Older Adults. Int. J. Environ. Res. Public Health.

[16] Okuyama J., Seto S., Fukuda Y., Funakoshi S., Amae S., Onobe J., Izumi S., Ito K., Imamura F. (2021). Mental health and physical activity among children and adolescents during the COVID-19 pandemic. Tohoku J. Exp. Med..

[17] Zahra A., Hassan M.S., Park J.H., Hassan S.-U., Parveen N. (2022). Role of Environmental Quality of Life in Physical Activity Status of Individuals with and without Physical Disabilities in Saudi Arabia. Int. J. Environ. Res. Public Health..

[18] Zahra A., Hassan S.U., Hassan M.S., Parveen N., Park J.-H., Iqbal N., Khatoon F., Atteya M.R. (2022). Effect of physical activity and sedentary sitting time on psychological quality of life of people with and without disabilities; A survey from Saudi Arabia. Front. Public Health..

[19] Washburn R.A., Zhu W., McAuley E., Frogley M., Figoni S.F. (2002). The physical activity scale for individuals with physical disabilities: Development and evaluation. Arch. Phys. Med. Rehabil..

[20] Alhumaid M.M., Said M.A., Adnan Y., Khoo S. (2024). Cross-Cultural Adaptation and Validation of the Arabic Version of the Physical Activity Scale for Individuals with Physical Disabilities in Saudi Arabia (PASIPD-AR). Healthcare.

[21] Diener E., Emmons R.A., Larsen R.J., Griffin S. (1985). The Satisfaction with Life Scale. J. Pers. Assess..

[22] Banville D., Desrosiers P., Genet-Volet Y. (2000). Translating questionnaires and inventories using a cross-cultural translation technique. J. Teach. Phys. Educ..

[23] Turney S. Pearson Correlation Coefficient (r)|Guide & Examples. Scribbr. Published 10 February 2024. https://www.scribbr.com/statistics/pearson-correlation-coefficient/.

[24] Alsubaie A.S. (2021). Associations between self-rated health, life satisfaction and physical activity among adolescents in Saudi Arabia. J. Med. Life.

[25] El Keshky M.E.S., Khusaifan S.J., Kong F. (2023). Gratitude and Life Satisfaction among Older Adults in Saudi Arabia: Social Support and Enjoyment of Life as Mediators. Behav. Sci..

[26] Pans M., Brewer B., Devís-Devís J. (2022). Life satisfaction in university students with disabilities: Differences by sociodemographic variables and associations with physical activity and athletic identity. J. Phys. Educ. Sport..

[27] Nicolaisen M., Strand B.H., Thorsen K. (2020). Aging with a physical disability, duration of disability, and life satisfaction: A 5-year longitudinal study among people aged 40 to 79 years. Int. J. Aging Hum. Dev..

[28] Emerson E., Llewellyn G. (2022). Disability among Women and Men Who Married in Childhood: Evidence from Cross-Sectional Nationally Representative Surveys Undertaken in 37 Low-and Middle-Income Countries. Int. J. Environ. Res. Public Health.

[29] Montgomery M. (2022). Reversing the Gender Gap in Happiness. J. Econ. Behav. Organ..

[30] Buecker S., Luhmann M., Haehner P., Bühler J.L., Dapp L.C., Luciano E.C., Orth U. (2023). The development of subjective well-being across the life span: A meta-analytic review of longitudinal studies. Psychol. Bull..

[31] Chan R.C. (2000). How does spinal cord injury affect marital relationship? A story from both sides of the couple. Disabil. Rehabil..

[32] Helliwell J.F., Layard R., Sachs J., De Neve J.-E. (2020). World Happiness Report 2020.

[33] Nemček D. (2016). Quality of life of people with disabilities: Differences in satisfaction with indicators and domains between active and inactive individuals. Phys. Act. Rev..

[34] Urchaga J.D., Guevara R.M., Cabaco A.S., Moral-García J.E. (2020). Life Satisfaction, Physical Activity and Quality of Life Associated with the Health of School-Age Adolescents. Sustainability.

[35] Martin Ginis K.A., Gee C.M., Sinden A.R., Tomasone J.R., Latimer-Cheung A.E. (2024). Relationships between sport and exercise participation and subjective well-being among adults with physical disabilities: Is participation quality more important than participation quantity?. Psychol. Sport. Exerc..

[36] Kim J., Kim M., Han A. (2018). Exploring the Relationship Between Types of Leisure Activities and Life Satisfaction, Health Perception, and Social Support among Korean Individuals with Physical Disabilities. Am. J. Health Behav..

[37] World Health Organization (2024). Assistive Technology. https://www.who.int/news-room/fact-sheets/detail/assistive-technology.

[38] Yang D., Zheng G., Wang H., Li M. (2022). Education, Income, and Happiness: Evidence from China. Front. Public Health.

[39] Tella R.D., MacCulloch R.J., Oswald A.J. (2003). The Macroeconomics of Happiness. Rev. Econ. Stat..

[40] Frey B.S., Stutzer A. (2002). What Can Economists Learn from Happiness Research?. J. Econ. Lit..

[41] Blanchflower D., Krueger A. (2010). International evidence of well-being. Measuring the Subjective Well-Being of Nations.

[42] Savitz D.A., Wellenius G.A. (2023). Can Cross-Sectional Studies Contribute to Causal Inference? It Depends. Am. J. Epidemiol..

[43] Brenner P.S., DeLamater J. (2016). Lies, Damned Lies, and Survey Self-Reports? Identity as a Cause of Measurement Bias. Soc. Psychol. Q..

[44] Dyrstad S.M., Hansen B.H., Holme I.M., Anderssen S.A. (2014). Comparison of self-reported versus accelerometer-measured physical activity. Med. Sci. Sports Exerc..

[45] Caramenti M., Castiglioni I. (2022). Determinants of Self-Perceived Health: The Importance of Physical Well-Being but Also of Mental Health and Cognitive Functioning. Behav. Sci..

